# Clinical significance of microRNA-125b and its contribution to ovarian carcinogenesis

**DOI:** 10.1080/21655979.2020.1814660

**Published:** 2020-09-06

**Authors:** Ya-Nan Bi, Jin-Ping Guan, Liming Wang, Ping Li, Feng-Xia Yang

**Affiliations:** a Department of Operating Room, The Affiliated Hospital of Qingdao University, Qingdao, Shandong, China; b Department of Surgery, The Affiliated Hospital of Qingdao University, Qingdao, Shandong, China; c Department of Gynecology, The Affiliated Hospital of Qingdao University, Qingdao, Shandong, China; d Department of Ultrasound, The Affiliated Hospital of Qingdao University, Huangdao, Shandong, China

**Keywords:** Ovarian cancer, metastasis, miR-125b, S100a4

## Abstract

The underlying mechanisms of recurrence and metastasis of epithelial ovarian cancer (EOC) are largely unknown. In the present study, we investigated the clinical significance of microRNA-125b (miR-125b) and its role in ovarian tumorigenesis and progression. Seventy patients of EOC and paired tissues were enrolled from 2015 to 2017. qRT-PCR was used to evaluate miR-125b expression in tumor tissues and EOC cell line. Gain-and-loss function of miR-125b was achieved to explore the changes in cell biological function. We found that miR-125b expression in EOC tissues, especially in the high-grade tissues (P < 0.001), was significantly lower compared to the matched adjacent noncancerous tissues and associated with pathological type, stage, and overall survival (P < 0.05). Upregulation of miR-125b promoted apoptosis and decreased cell survival rate and migration, and vice versa *in vitro*. Mechanistically, miR-125b negatively regulated S100A4, a metastasis-associated protein. MiR-125b overexpression significantly decreased tumor growth and inhibited lung metastasis *in vivo*. Our results supported that miR-125b contributes to the progression of EOC by targeting S100A4. It potentially acts as a potential biomarker and therapeutic target of EOC.

## Graphical abstract

**Figure:**
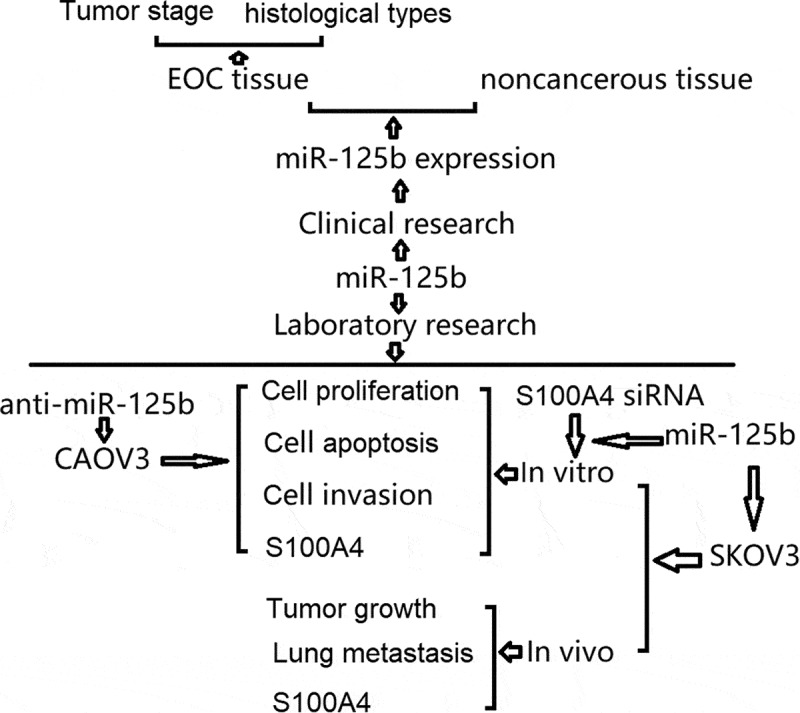

## Introduction

Epithelial ovarian cancer (EOC) is the deadliest of gynecologic cancers and the leading cause of cancer-related death [1]. It is often diagnosed at an advanced stage with extensive local and systemic spread and poor survival [2]. Currently, there is no proven tool for early EOC detection due to nonspecific symptoms in its early stage [3].

Standard therapy for EOC is of the combined surgical debulking and platinum‐based chemotherapy, but less than half respond [4]. Despite novel chemotherapeutic regimes and targeted therapies have been used in clinic, there have been no significant improvements in clinical outcomes, and the 5-year overall survival rates are less than 45% [5,6]. Therefore, improved biologic marker for diagnosis, treatment, and prognosis of EOC is urgently needed.

MicroRNAs (miRNAs) are small non-coding RNAs important in gene regulation [7], by which to regulate cell function such as the cell cycle, proliferation and apoptosis, et al. [8,9]. Emerging evidence has demonstrated that microRNAs (miRNAs) downregulation is involved in cancer metastases [10,11]. A lot of miRNAs have been considered as the promising markers for cancer diagnosis and prognosis as well as monitoring the treatment response, particularly in ovarian cancer [12].

Abnormal microRNA-125b (miR-125b) expression has been demonstrated in numerous human cancers. For example, miR-125b was downregulated in esophageal squamous cell carcinoma [13], bladder cancer [14], primary glioblastoma [15] and hepatocellular carcinoma [16] and functioned as a tumor suppressor. Also, miR-125b was upregulated in anaplastic thyroid cancer [17], gastric cancer [18], and acute myeloid leukemia [19] and functioned as a tumor promoter. Zhu et al. [20] reported that serum miRNA-125b levels were significantly enhanced in early stages I and II of ovarian cancer patients, and higher miR-125b expression was positively related with progression-free survival and marginally and overall survival. Guan et al. [21] reported that enforced miR-125b expression induced cell cycle arrest and reduced cell proliferation and clonal formation in EOC cells. Yang et al. [22] reported that enforced miR-125b blocked ascites-induced cell migration in EOC cells. However, the clinical significance and the function of miR-125b in patients with EOC and EOC cells are rarely investigated.

S100A4 is specific S100 family metastasis-related protein, which is upregulated in many cancer tissues and cell lines, where it controls cell proliferation, differentiation and metastasis [23,24]. Numerous studies have found that miRNAs play crucial roles during human tumorigenesis and development by regulation of S100A4, such as MicroRNA-187 [25], miR-149-3p [26] and miR-296 [27].

Here, we confirmed that miR-125b was lowly expressed in EOC tissues, and low miR-125b expression was correlated with poor prognosis in patients with EOC. Furthermore, enforced miR-125b expression has an obvious inhibitory effect on the growth, invasion and metastasis of EOC cells *in vivo* and *in vitro*. Additionally, the effect of miR-125b on the biological behavior of EOC cells may be achieved by regulating S100A4 expression.

## Materials and methods

### Patients and clinical data

Seventy paired samples of human EOC and their matched adjacent noncancerous tissues were collected at the time of surgery between July 2015 and July 2017 from the affiliated hospital of Qingdao University. Upon resection, the surgical specimens were placed immediately in liquid nitrogen and stored at −80°C in the refrigerator. All participating patients obtained informed consent. All procedures used here have been approved by the review board for human research of the affiliated hospital of Qingdao University. The final diagnosis was established by two pathologists. Patient’s age ranges from 16-year to 68-year and the age was 53.5 (27–83) years. The number of the patients in the different tumor stage was I (n = 3), II (n = 5), III (n = 41), and IV (n = 21), respectively; The number of the patients in the different histology was: Serous (n = 58), Mucinous (n = 4), Endometrioid (n = 6) and Clear cell (n = 2). Inclusion criteria include 1. Complete clinical data; 2. Definite pathologic diagnosis; 3. Signed informed consent; 4. No prior radiochemo-, or immunotherapy; 5. Other than the ovarian tumor, the patient’s past medical history was not significant.

### Cell lines and cell cultures

SKOV3, A2780, SKOV3ip1, OVCAR 5 and CAOV3 cell lines were obtained from the Type Culture Collection Center of Chinese Academic of Science (Shanghai, China), and cultured in Dulbecco’s modified Eagle’s medium (DMEM), supplemented with 10% fetal bovine serum (FBS) in 37°C with 5% CO_2_ incubator.

### In vitro *transfection*

The miR-125b mimics (miR-125b), miR-125b inhibitors (anti-miR-125b) and their corresponding control miR-NC and anti-miR-NC were synthesized by Ambion (Applied Biosystems, CA, US). EOC cells were seeded in a 6-well plate (1 × 10^6^ cells per well) and cultured for 24 h to a confluency of 60–80%. Then the cells were transfected with miR-125b mimics, miR-125b inhibitors or their corresponding controls for 48 h to a final concentration of 100 nM using the Neon® Transfection System (Life Technologies) (1050 v, 2 pulse, 30 a) according to the manufacturer’s instructions. Similar conditions were used to transfect EOC cells with S100A4 siRNA or control siRNA (si CN) at a final concentration of 50 nM even in cotransfection experiments with miRNAs inhibitors.

### Recombinant vector transfection

pEGFP-S100A4 and control pEGFP plasmids were synthesized and purchased from Genechem (Shanghai, China). Transfection of the plasmids in the EOC cells was performed using the Neon® Transfection System as per the manufacturer’s protocols (Invitrogen).

### Stable miR-125b transfection

The recombinant lentivirus encoding mir-125b (Lv-mir-125b) and control miR-NC (Lv-NC) were obtained from Genecheme (Shanghai, P.R. China). All lentiviral particles contain EGFP gene. Colonies with GFP expression were selected by limiting dilution assay of 96-well plate to expand culture. Finally, EOC subline with miR-125b overexpression was established for further investigation.

### Quantitative RT-PCR

Total RNA was isolated from EOC tissues and cell cultures using mirVana miRNA Isolation Kit (Ambion, Austin, TX) as per the manufacturer’s recommendations. PrimeScript™ RT Master Mix Kit (Takara Biotechnology Co., Ltd.) was used to synthesize the cDNA. The RT-qPCR was performed on samples from tissues and cultured cells using Biosystems PCR750 system as the manufacturer’s instruction. The sequences of miR-125b inhibitors used are as follows: 5′-ACGGATTCTCGGGAAAATCGAGC-3′. The scrambled antimiR sequence was 5′-CGCCTGAATGAATAACCGACCAG-3′. The relative gene expression was normalized to *U6*. The miR-125b expression was calculated using the delta-delta Ct method (2^−ΔΔ*C*^_T_). All results were performed in triplicate.

### Western blotting

The cultured EOC cells were harvested and lysed in RIPA buffer (Amresco N653) at 4°C for 40 min and then separated by electrophoresis, incubated with anti-S100A4 and anti-actin (Cell Signaling 3285, Shanghai, China).

### Cell apoptosis assay

The harvested cells (1 × 10^6^) were digested with a trypsin solution and washed with PBS and resuspended in 500 μl binding buffer. Cells were finally resuspended in 400 μl of Binding Buffer 1X and 40 μg/mL (100 μl) PI and 100 lg/mL RNaseA (Sigma-Aldrich) and incubated for 1 min before analyzing with the flow cytometer. The data were analyzed on the BD FACSCalibur cell sorting system and repeated three times.

### MTT assay

Cells in the logarithmic growth phase were plated in 96-well plates. After 24 h, the cells (3000/well) were transfected with miR-125b or anti-miR-125b or its control or co-transfection with S100A4 siRNA for 24–96 h using Lipofectamine RNAiMAX as the manufacturer’s instruction. Following the addition of 20 μl of MTT reagent (5 mg/ml) to each well and incubation for 4 h, the medium was removed and replaced with 150 μl of dimethylsulfoxide in each well. The absorbance was measured on a microplate reader at a wavelength of 540 nm.

### Colony formation assay

Transfected cells (1000 cells/well) were seeded into a 6-well plate and cultured for 10 days. Then, the cells were fixed and stained with crystal violet. Colonies with a diameter >50 cells were counted and the survival fractions were calculated. The experiment was repeated three times.

### Transwell invasion assay

The parental and transfected cells (2 × 10^5^) were seeded in Matrigel-coated invasion chambers (Invitrogen) and incubated for 24 h at 37°C. Cells on the top of inserts were swabbed. The inserts were then fixed and stained, and the fluorescent signal was measured. The experiments were repeated three times.

### In vivo *studies*

For tumor growth experiments, 6 × 10^6^ SKOV3 cells [SKOV3 subline stably miR-125b (SKOV3-LV-miR-125b mimic) and its control line (SKOV3-LV-NC)] were subcutaneously injected into the flank of mice and measured the tumor volume with calipers every 5 days, and determined using the following formula: Tumor volume = (width^2^ × length)/2.

For lung metastasis experiments, the SKOV3-LV-miR-125b mimic or SKOV3-LV-NC cells (5 × 10^5^) were injected into the 6-week-old male nude mice through the tail vein. The mice were sacrificed, and lung surface tumor foci were counted 4 weeks after injection. Tumor and metastasis samples were collected for RT-qPCR or immunohistochemistry assay. Experiments were performed under the approval of the affiliated hospital of Qingdao University.

### Statistical analysis

All data are presented as the mean ± SD. Data were compared using t-test or one-way analysis of variance (ANOVA). Analysis for significance was performed using SAS 6.12 statistical software (SAS Institute). Statistical significance was determined by *p < 0.05. **p < 0.01.

## Results

### miR-125b expression is correlated with tumor stage

**Figure 1.**
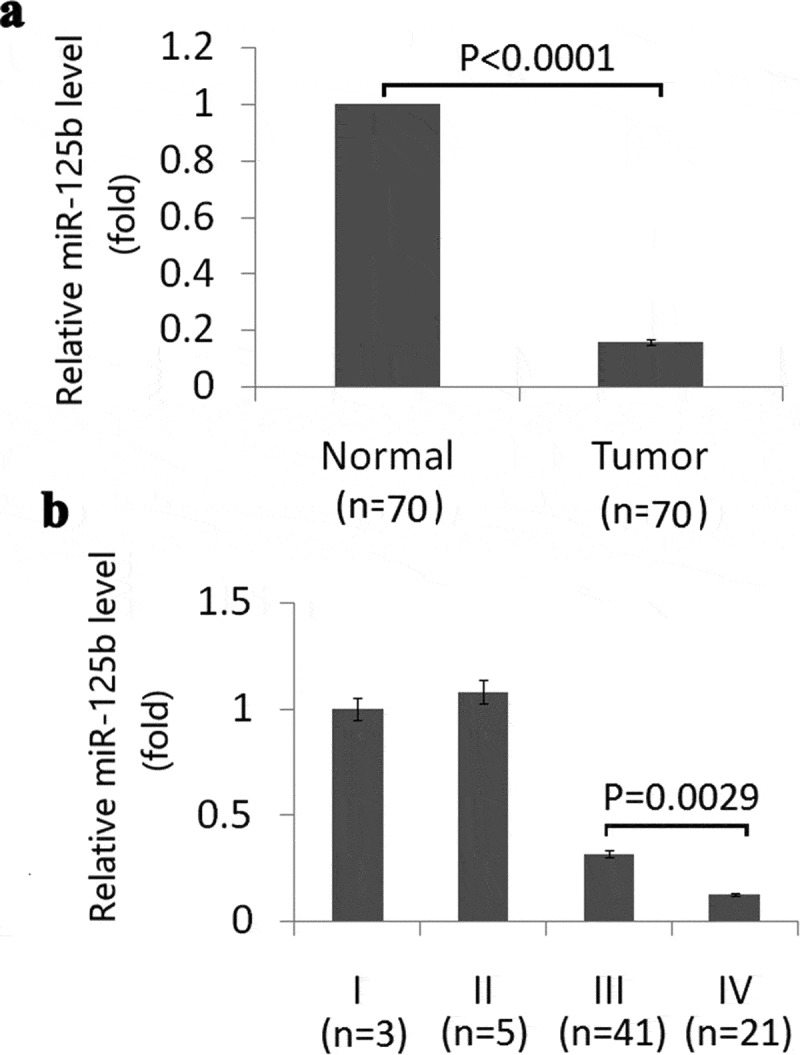
Expression of miR-125b in EOC tissues and its relation with tumor stage. (a), Expression of miR-125b in EOC tissues and matched adjacent noncancerous tissues by qRT-PCR analysis (p < 0.0001). (b), Expression of miR-125b in different stages in EOC tissues by qRT-PCR analysis (p = 0.0029).

We first detected miR-125b expression in EOC tissues and their matched adjacent noncancerous tissues from 70 patients of human EOC. As showed in Figure 1(a), miR-125b was downexpressed in EOC tissues compared to the matched adjacent noncancerous tissues.

No significant differences were found among different histological types of EOC (P = 0.0672). But the expression of miR-125b reduced significantly in tumors of stage IV compared with the tumors of stage III (p = 0.0029) (Figure 1(b)), indicating that miR-125b overexpression was involved in metastatic progression.

### Enforced miR-125b expression on cell viability and apoptosis

**Figure 2.**
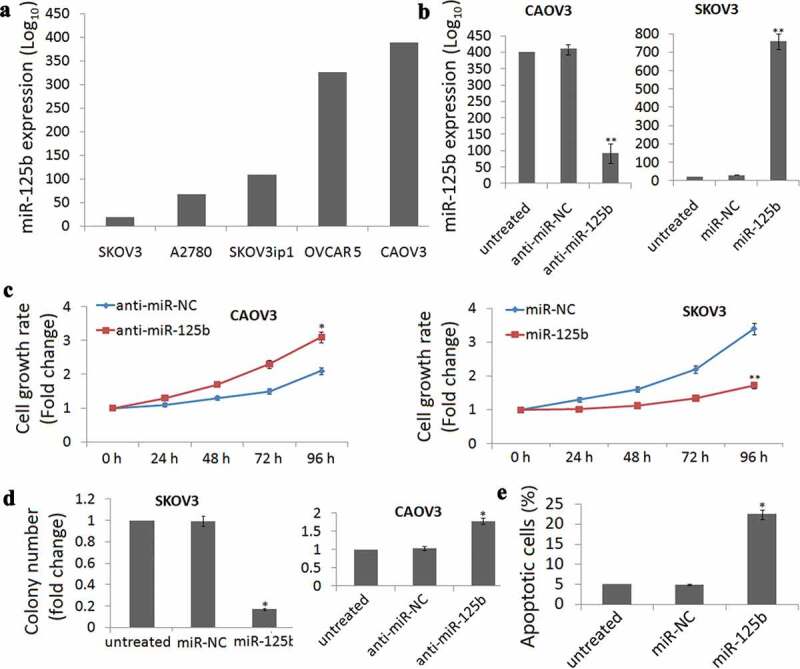

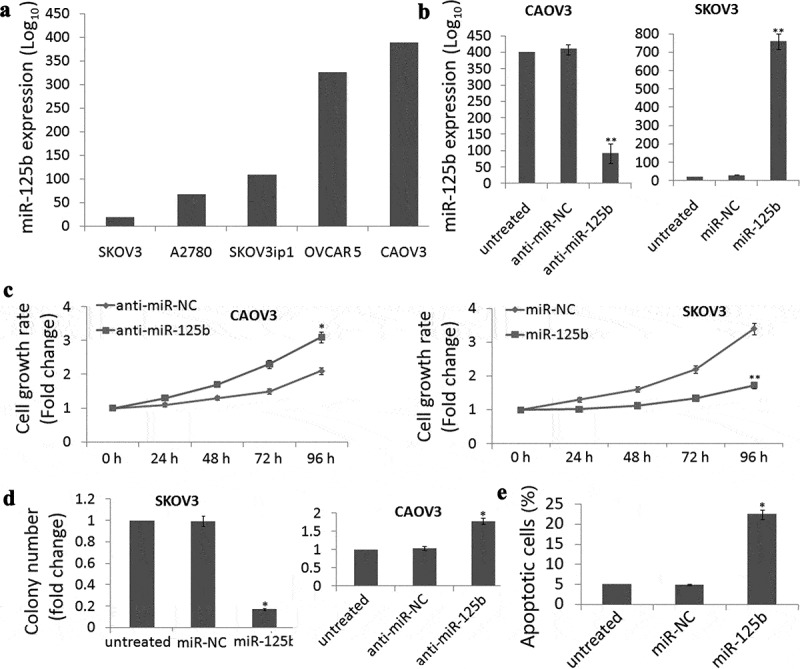
miR-125b decreases OC cell survival, proliferation and induces cell apoptosis *in vitro*. (a), qRT-PCR analysis showing different levels of miR-125b expression in the ovarian cancer cell lines SKOV3, A2780, SKOV3ip1, OVCAR 5 and CAOV3. (b), qRT-PCR analysis showing lower expression of miR-125b in CAOV3 cells after transfection with anti-miR-125b. (c), qRT-PCR analysis showing higher expression of miR-125b in SKOV3 cells after transfection with miR-125b. (d), CAOV3 cells transfected with anti-miR-125b had signiﬁcantly increased survival by MTT assay, and SKOV3 cells transfected with miR-125b had signiﬁcantly decreased survival by MTT assay. (D), CAOV3 cells transfected with anti-miR-125b increased the number of colonies, and SKOV3 cells transfected with miR-125b decreased the number of colonies; (e), SKOV3 cells transfected with miR-125b increased the number of apoptosis.*P < 0.05;**P < 0.01.

miR-125b expression was detected in SKOV3, A2780, SKOV3ip1, OVCAR 5 and CAOV3 cells by qRT-PCR assay and found that different miR-125b expressions were observed in the five EOC cell lines (Figure 2(a)). Among the five cell lines, highest expression was found in the CAOV3 cells and lowest expression was found in the SKOV3 cells. So we used CAOV3 and SKOV3 cells for the study.

CAOV3 cells were transfected with anti-miR-125b to decrease miR-125b expression (Figure 2(a)), while SKOV3 cells were transfected with miR-125b to increase miR-125b expression (Figure 2(b)). MTT assay demonstrated that cell viability was time-dependent increase in the CAOV3 cells (Figure 2(c)) and time-dependent decrease in the SKOV3 cells (Figure 2(c)) after 24 h-96 h cell transfection.

Colony formation assays were also conducted and shown that targeting miR-125b increased the colonies in CAOV3 cells and decreased the colonies in the SKOV3 cells (Figure 2(d)). Further experiments demonstrated that enforced miR-125b expression significantly induced cell apoptosis in the SKOV3 cells (*P*= 0.018, Figure 2€).

### *Enforced miR-125b expression inhibits cell invasion* in vitro

**Figure 3.**
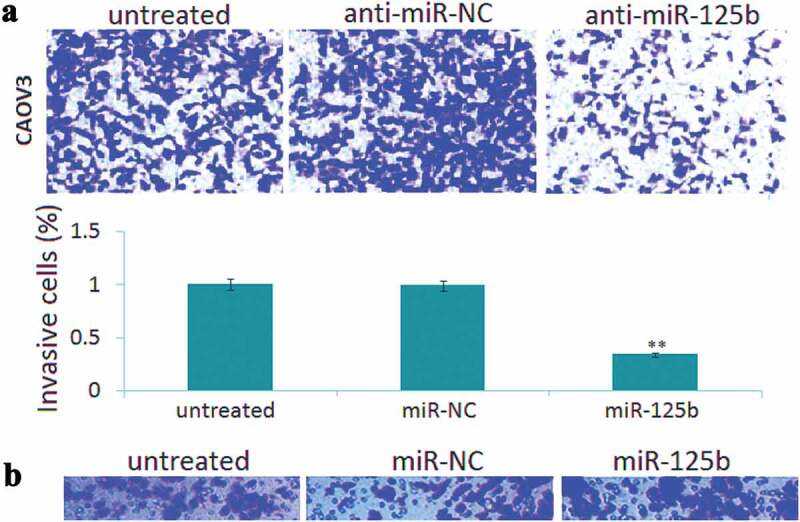

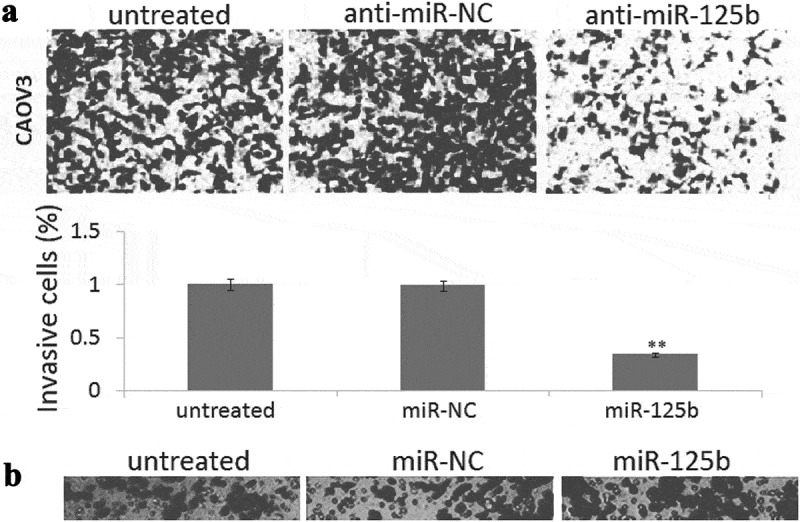
miR-125b overexpression decreases ovarian cancer cell invasion potential in vitro. (a), Representative photographs of invaded SKOV3 cells in different groups. (b), Representative photographs of invaded CAOV3 cells in different groups. *P< 0.05.

Transwell invasion assay showed that enforced mir-125b expression led to 48% increase of invasive cells in the SKOV3 cells (Figure 3(a)), and targeting miR-125b led to 53% reduction of invasive cells in the CAOV3 cells (Figure 3(b)). No significant difference of cell invasion was found among the control groups (Figure 3(a,b)).

### miR-125b functions in EOC cells via regulating S100A4 expression

**Figure 4.**
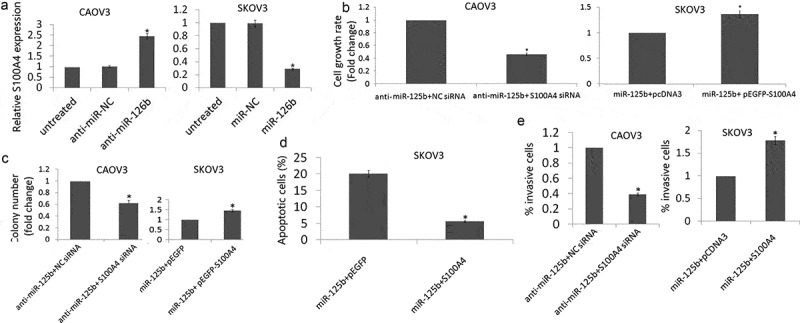
Effect of S100A4 on miR-125b-induced cell proliferation, apoptosis and cell invasion. (a), Western blot was used to detect S100A4 protein in CAOV3 cells transfected with anti-miR-125b or anti-NC, and SKOV3 cells transfected with miR-125b mimic or miR-NC. (b), Cell proliferation was detected by MTT in CAOV3/anti-miR-125b cells transfected with S100A4 siRNA on NC siRNA, or in SKOV3/miR-125b cells transfected with pEGFP-S100A4 or pEGFP; (c), Colony formation assay was used to detect cell growth in CAOV3/anti-miR-125b cells transfected with S100A4 siRNA on NC siRNA, or in SKOV3/miR-125b cells transfected with pEGFP-S100A4 or pEGFP. (d), Cell apoptosis was detected in SKOV3/miR-125b cells transfected with pEGFP-S100A4 or pEGFP using Flow cytometry analysis. E,Cell invasion was detect in CAOV3/anti-miR-125b cells transfected with S100A4 siRNA on NC siRNA, or in SKOV3/miR-125b cells transfected with pEGFP-S100A4 or pEGFP.*P< 0.05.

Low S100A4 protein expression was observed in the CAOV3 cells, and high S100A4 protein expression was observed in the SKOV3 cells by western blot assay. Knockdown of miR-125b in CAOV3 cells induced S100A4 expression, and enforced miR-125b expression inhibited S100A4 expression in SKOV3 cells (Figure 4(a)).

On the other hand, co-transfection with anti-miR-125b and S100A4 siRNA partially blocked the anti-miR-125b-induced pro-proliferation and pro-growth effects in the CAOV3 cells (Figure 4(b,c)) and pro-invasive effects (Figure 4(e)). In addition, co-transfection with miR-125b and pEGFP-S100A4 partially blocked miR-125b-induced pro- apoptotic effect in the SKOV3 cells (Figure 4(d)) and anti- proliferation effects (Figure 4(b,c)) and anti-invasive effects (Figure 4(e)).

### *Enforced miR-125b reduces tumor growth and lung metastasis* in vivo

**Figure 5.**
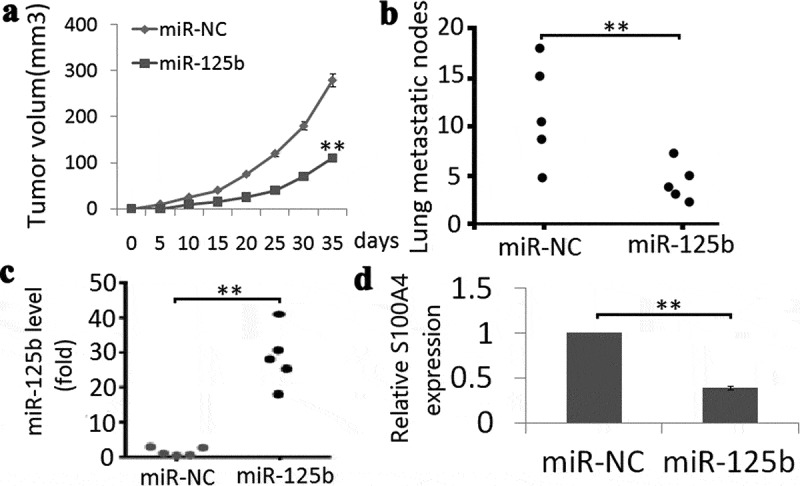
Effect of miR-125b on tumor growth and lung metastasis in vivo. (a), Volumes of all tumors were detected every 5 days; (b), Quantification d of macroscopic metastatic nodules on the lung surface. (c), miR-125b expression was detected in two groups by Qrt-PCR assay; (d), S100A4 protein expression was detected in two groups by Western blot assay. Vs miR-NC, ** P < 0.01.

We first subcutaneously injected SKOV3-Lv-miR-125b mimic or SKOV3-Lv-miR-NC cells into the right flank of nude mice. The results showed that miR-125b overexpression inhibited tumor growth 28 days after tumor formation (Figure 5(a); *P* < 0.01). We then injected SKOV3-Lv-miR-125b mimic or SKOV3-Lv-miR-NC cells into nude mice via the tail vein to observe tumor metastasis. Few lung metastatic nodules were observed in SKOV3-Lv-miR-125b mimic cells after 4 weeks tail injection compared to the controls (Figure 5(b); *P* < 0.01), indicating that miR-125b overexpression reduces tumor growth and lung metastasis.

Expression of miR-125b and S100A4 was analyzed using qRT-PCR and western blotting in tumor tissues, respectively. The results showed that miR-125b expression was higher (Figure 5(c)) and S100A4 was lower (Figure 5(d)) in the SKOV3-LV-miR-125b mimic tumor tissues compared with the SKOV3-LV-miR-NC tumor tissues.

## Discussion

Ovarian cancer, diagnosed at a late stage mainly due to the lack of early detection methods, is the highest lethal gynecological cancers, with a 5-yr survival rate of <30% [28]. Numerous studies have shown that miRNAs may be the valuable diagnostic and prognostic markers for cancers, including EOC [12,29].

MiR-125 overexpression has been reported in tissues of gliomas and prostate cancer [30,31], while lower miR-125 expression was reported in breast and gastric cancer tissues [32,33]. Serum miR-125b levels were found to be a useful biomarker to predict prognosis and the responses to chemotherapy in patients with EOC [34]. However, the miR-125b expression in EOC tissues and its effect on EOC has not been documented. Here, we found that miR-125b is downexpressed in EOC tissues, and lower miR-125b expression was relative with high tumor stage. Therefore, miR-125b downexpression may be considered as the poor prognosis and unfavorable outcome for patients with EOC.

Accumulated evidence showed that overexpression of miRNAs using synthetic miRNAs mimics or targeting miRNAs using molecules has therapeutic potential for cancers [35–37]. In human invasive breast cancer, enforced miR-125b expression inhibited cell growth and lung metastasis *in vitro* and *in vivo* [38]. However, in glioma, enforced miR-125b expression promotes growth and clone formation, and protects the glioma cells from apoptosis *in vitro* and *in vivo* [39]. In this study, miR-125b overexpression was observed to induce EOC cell apoptosis and inhibited tumor growth *in vitro* and *in vivo*. Furthermore, miR-125b overexpression inhibits invasion *in vitro* and lung metastasis *in vivo*. Therefore, it can be assumed that miR-125b may be the potential target for the treatment of EOC.

Epithelial–mesenchymal transition (EMT) represents a cell biological program and regulates cell invasion and metastasis in cancers, and miRNAs regulates EMT-associated signaling genes [40]. As a metastasis-associated protein and marker of the EMT, S100A4 contributes to the progression of several types of cancer [41]. Giving its important role in metastasis, S100A4 may be a potential target for cancer therapy. S100A4 overexpression has reported to be a marker of metastasis, advanced TNM stages and poor prognosis in patients with EOC [42]. S100A4 could also be regulated by miRs. For example, miR-187-3p inhibits the metastasis and EMT in hepatocellular carcinoma and osteosarcoma by targeting S100A4 [25,26]. S100A4 mediated miR-149-3p-induced anti-metastatic effects of bladder cancer cells in vitro and in vivo [43]. In this study, we observed that enforced miR-125b expression inhibits invasion and metastasis of EOC cells by targeting S100A4 expression. However, targeting miR-125b increased the invasive ability of EOC cells by S100A4 upregulation.

Targeting S100A4 siRNA has reported to decrease proliferation and induce apoptosis in anaplastic thyroid cancer (ATC) cells in vitro and in vivo, supporting that S100A4 is responsible for increased survival [43]. This study indicated that targeting S100A4 reversed miR-125b-induced cell apoptosis and growth inhibition, and vice versa, suggesting the miR-125b inhibits OC cell growth by S100A dependent pathway.

## Conclusion

The study showed that miR-125b is lowly expressed in EOC tissues. miR-125b downregulation contributes to the progression of EOC. Enforced miR-125b expression inhibited cell invasion, proliferation, tumor growth and metastasis. MiR-125b-mediated function in EOC cells was by targeting S100A4 expression. These data indicated that miR-125b may be the potential diagnostic and therapeutic target for EOC.

## Highlights

1. miR-125b was overexpressed in EOC tissues.
2. miR-125b expression is associated with pathological type, stage, and overall survival in patients with EOC.
3. MiR-125b overexpression decreased tumor growth and inhibited lung metastasis by targeting S100A4
